# Circulating microRNAs May Serve as Biomarkers for Hypertensive Emergency End-Organ Injuries and Address Underlying Pathways in an Animal Model

**DOI:** 10.3389/fcvm.2020.626699

**Published:** 2021-02-12

**Authors:** Knut Asbjørn Rise Langlo, Gustavo Jose Justo Silva, Tina Syvertsen Overrein, Volker Adams, Ulrik Wisløff, Håvard Dalen, Natale Rolim, Stein Ivar Hallan

**Affiliations:** ^1^Department of Circulation and Medical Imaging, Faculty of Medicine and Health Sciences, Norwegian University of Science and Technology, Trondheim, Norway; ^2^Department of Nephrology, Clinic of Medicine, St. Olav's Hospital, Trondheim University Hospital, Trondheim, Norway; ^3^Institute for Experimental Medical Research, Oslo University Hospital and University of Oslo, Oslo, Norway; ^4^Division of Pathology and Medical Genetics, Department of Laboratory Medicine, St. Olav's Hospital, Trondheim University Hospital, Trondheim, Norway; ^5^Department of Cardiology, Heart Center Dresden, TU Dresden, Dresden, Germany; ^6^School of Human Movement & Nutrition Sciences, University of Queensland, Brisbane, QLD, Australia; ^7^Clinic of Cardiology, St. Olav's Hospital, Trondheim University Hospital, Trondheim, Norway; ^8^Department of Medicine, Levanger Hospital, Nord-Trøndelag Hospital Trust, Levanger, Norway; ^9^Department of Clinical and Molecular Medicine, Faculty of Medicine and Health Sciences, Norwegian University of Science and Technology, Trondheim, Norway

**Keywords:** hypertensive emergency, hypertensive encephalopathy, thrombotic microangiopathy, heart failure with preserved ejection fraction, endothelial dysfunction, microRNA, pathway prediction

## Abstract

There is an incomplete understanding of the underlying pathophysiology in hypertensive emergencies, where severely elevated blood pressure causes acute end-organ injuries, as opposed to the long-term manifestations of chronic hypertension. Furthermore, current biomarkers are unable to detect early end-organ injuries like hypertensive encephalopathy and renal thrombotic microangiopathy. We hypothesized that circulating microRNAs (c-miRs) could identify acute and chronic complications of severe hypertension, and that combinations of c-miRs could elucidate important pathways involved. We studied the diagnostic accuracy of 145 c-miRs in Dahl salt-sensitive rats fed either a low-salt (*N* = 20: 0.3% NaCl) or a high-salt (*N* = 60: 8% NaCl) diet. Subclinical hypertensive encephalopathy and thrombotic microangiopathy were diagnosed by histopathology. In addition, heart failure with preserved ejection fraction was evaluated with echocardiography and N-terminal pro-brain natriuretic peptide; and endothelial dysfunction was studied using acetylcholine-induced aorta ring relaxation. Systolic blood pressure increased severely in animals on a high-salt diet (high-salt 205 ± 20 mm Hg vs. low-salt 152 ± 18 mm Hg, *p* < 0.001). Partial least squares discriminant analysis revealed 68 c-miRs discriminating between animals with and without hypertensive emergency complications. Twenty-nine c-miRs were strongly associated with hypertensive encephalopathy, 24 c-miRs with thrombotic microangiopathy, 30 c-miRs with heart failure with preserved ejection fraction, and 28 c-miRs with endothelial dysfunction. Hypertensive encephalopathy, thrombotic microangiopathy and heart failure with preserved ejection fraction were associated with deviations in many of the same c-miRs, whereas endothelial dysfunction was associated with a different set of c-miRs. Several of these c-miRs demonstrated fair to good diagnostic accuracy for a composite outcome of hypertensive encephalopathy, thrombotic microangiopathy and heart failure with preserved ejection fraction in receiver-operating-curve analyses (area-under-curve 0.75–0.88). Target prediction revealed an enrichment of genes related to several pathways relevant for cardiovascular disease (e.g., mucin type O-glycan biosynthesis, MAPK, Wnt, Hippo, and TGF-beta signaling). C-miRs could potentially serve as biomarkers of severe hypertensive end-organ injuries and elucidate important pathways involved.

## Introduction

Hypertensive emergency is a life-threatening condition occurring when untreated hypertension exceeds the limits of organ blood flow autoregulation, typically with acutely increased blood pressure to 200/120 mmHg or higher (1). Subsequently, this can damage the brain, kidney, and heart, and the blood pressure needs to be controlled immediately in such patients to prevent further end-organ injury (1, 2). However, no biomarkers are currently available to support initial management of these patients (3, 4).

Hypertensive encephalopathy (HE) often presents with non-specific symptoms of mild cognitive dysfunction and headache due to cerebral edema, and magnetic resonance imaging (MRI) may reveal white matter lesions and microbleeds (5–8). Furthermore, thrombotic microangiopathy (TMA) may injure the glomerular capillaries leading to rapidly progressive renal failure. TMA lesions are less reversible than the acute tubular injury frequently seen with general hypoxia or nephrotoxins, emphasizing the need for urgent treatment (9). Less acutely, severe untreated hypertension often leads to chronic heart failure with preserved ejection fraction (HFpEF). One hypothesized link between these end-organ injuries is endothelial dysfunction (ED) combined with a chronic inflammatory state (10, 11). If end-organ injuries could be revealed at an earlier stage, selection of the right patients for early and intensified treatment would improve. However, serum creatinine levels starts to rise 1–2 days after the acute kidney injury, MRI scanning of the brain is expensive and often not immediately available, and current biomarkers for HFpEF lack sensitivity and specificity (12, 13). Development of new biomarkers is hampered by our incomplete understanding of the underlying mechanisms. MicroRNAs (miRs) may serve multiple purposes in this setting. MiRs are a class of short non-coding RNA molecules that inhibit transcription, translation, or degradation of target genes (14). Tissue-derived miRs have been associated with both hypertension and cardiovascular disease (15–20), acting as essential intracellular mediators of cardiac function (15). The knowledge about freely circulating miRs (c-miRs) is rapidly expanding, although their biological importance remains to be established (21–23). Studies indicate that c-miRs could be sensitive and specific biomarkers in several diseases, with the important advantage of minimal invasive sampling, reproducibility, and a rapidly available test result (14, 24–27).

Circulating miRs are not well-studied in the hypertensive emergency state, so we aimed to investigate c-miRs as potential biomarker candidates for the hypertensive end-organ injuries TMA and HE in Dahl/Salt-Sensitive (SS) rats on a high- or low-salt diet. Further, we investigated c-miRs as biomarkers for HFpEF as a chronic complication of severe long-standing hypertension and ED as a possible link between different end-organ injuries in severe hypertension. miR databanks were used to elucidate potential pathways through which the c-miRs could exert their effects.

## Materials and Methods

### Animal Model of Hypertensive Emergency

This investigation is based on a rodent model of severe hypertension, previously described in the OptimEx Study (28, 29). In brief, female Dahl/salt-sensitive (Dahl/SS) rat (SS/JrHsdMcwiCrl, Charles River Laboratories, Wilmington, NC, USA) given a high-salt diet is a well-established animal model for severe hypertension complicated with brain, heart, kidney, and other organ failures (30–32). Our cohort started out with 80 rats divided into a low-salt diet (LS, 0.3% NaCl, *n* = 20) or a high-salt diet (HS, 8% NaCl, *n* = 60), starting at 9 weeks of age, to induce hypertensive end-organ injuries. Dahl/SS on a LS diet typically develop mild to moderate hypertension. Animals without signs of severe hypertensive complications were terminated at 29–30 weeks of age in a random order, and group status were blinded to the investigators. According to protocol, animals with severe symptoms were terminated and not included in our study of early biomarkers. The animal experiments were approved by the Norwegian Food Safety Authority (FOTS 5407) and was in accordance with ≪The Norwegian Regulation on Animal Experimentation≫ and ≪Directive 2010/63/EU on the protection of animals used for scientific purposes≫. All included Dahl/SS rats were evaluated together for the presence or absence of four hypertensive outcomes: HE, TMA, HFpEF, and ED.

### Clinical Characteristics

Non-invasive blood pressure was measured on several occasions in conscious rats restrained in a chamber with a controlled temperature between 32 and 34°C. Blood pressure was measured using a tail-cuff plethysmography (Kent Scientific Corporation, Torrington, CT, USA). Urine was sampled during metabolic cage observations for 24 h at the end of the study. Blood was sampled *via* cardiac catheterization before the animals were terminated. Creatinine was analyzed enzymatically. Albumin was analyzed immunologically. Both performed at laboratory services, St. Olav's Hospital.

### Definition of Outcomes

Transthoracic echocardiography was performed and analyzed using a Vevo 2100 system (FujiFilm VisualSonics, Toronto, ON, Canada). Animals were lightly anesthetized (1.5–2% isoflurane) but spontaneously breathing. All calculated parameters were automatically computed by the Vevo 2100 standard measurement package. Left ventricular (LV) ejection fraction (LVEF) were calculated from M-mode recordings in parasternal short axis. From the pulsed wave Doppler spectral waveforms, we measured the peak early- and late-diastolic trans-mitral velocities (E and A waves). From the tissue Doppler spectral waveforms, we measured the early-diastolic myocardial relaxation velocity (e′) and calculated E/e′ ratio. Both were obtained from the apical four-chamber view. Left atrial dimensions (LAD) were also measured from apical four-chamber view and corrected for body weight (BW). All measurements were performed outside the respiration peaks and obtained in triplicate. Complementary to the echocardiographic parameters, we used N-terminal pro-brain natriuretic peptide (NT-proBNP) for the HFpEF classification. NT-proBNP levels were determined *via* enzyme-linked immunosorbent assay (ELISA) according to manufacturer's specification (CUSABIO, China). The definition of HFpEF still varies among human guidelines (33, 34), and for animals there are no guidelines at all (35). In our study, HFpEF was defined as a combination of four parameters, E/A, E/e′, LAD/BW ratios, and NT-proBNP, above the 75th percentiles of the LS animals, combined with a left ventricular ejection fraction (EF) >50% (29).

Endothelium-dependent vasodilation was tested *in vitro* by stimulation of aortic rings with increasing concentrations of acetylcholine (28). In the same manner as with HFpEF, we operationalized the dichotomous definition of endothelial dysfunction as cases needing acetylcholine concentrations higher than the 75th percentile obtained in the LS group to achieve a 50% dilatation of the aortic rings (EC_50_).

**Figure 1:**
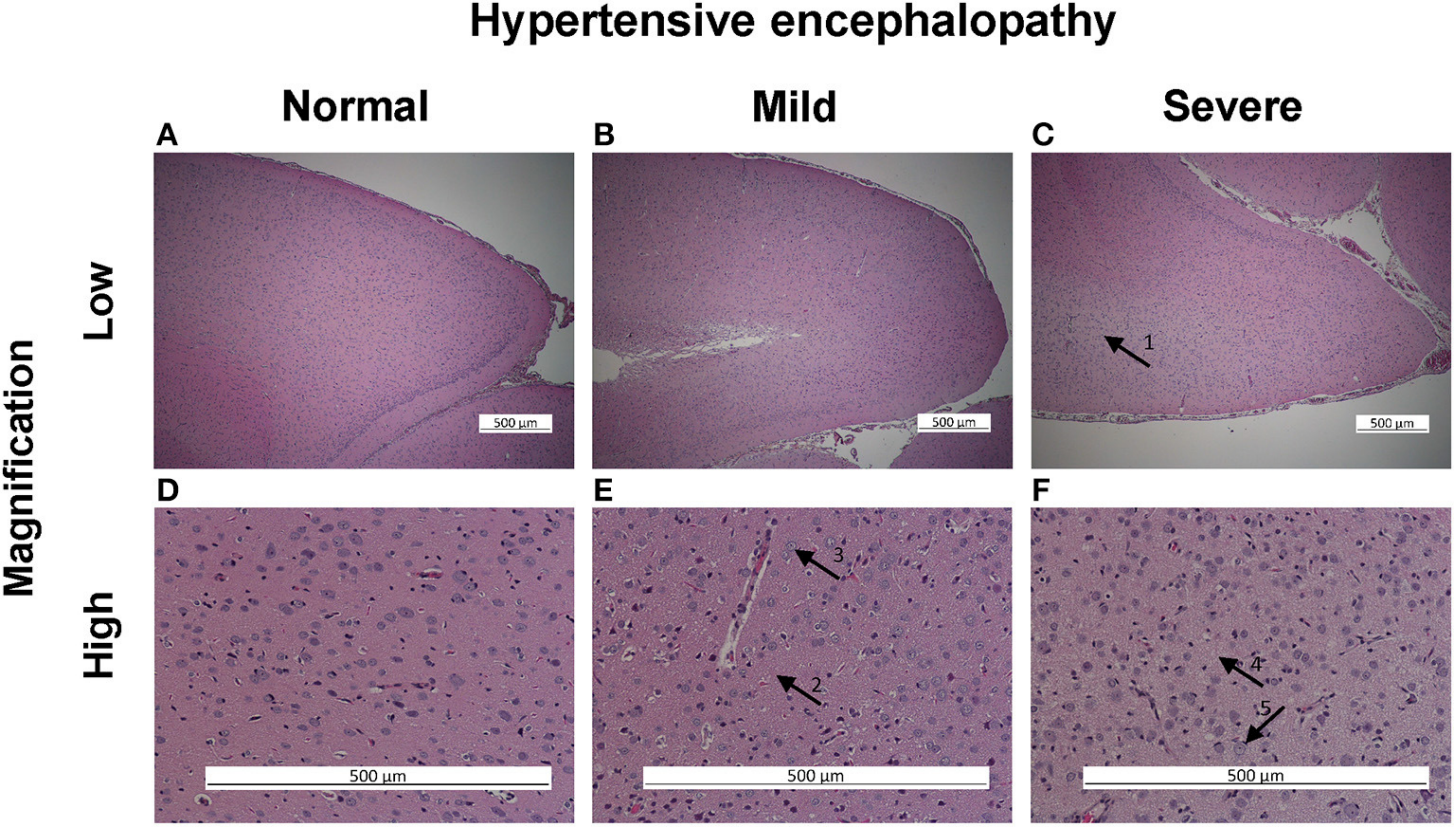
Hypertensive encephalopathy histological assessment in PAS staining, posterior cerebrum. In low magnification illustrating no **(A)**, mild **(B)**, and severe **(C)** hypertensive encephalopathy (HE). Severe HE characterized by central pale staining and looser ground mesh (arrow 1). In high magnification illustrating no **(D)**, mild **(E)**, and severe **(F)** changes of HE. Mild changes characterized by somewhat looser ground mesh (arrow 2) and some vacuolated nuclei (arrow 3). Severe changes characterized by very loose ground mesh (arrow 4) and vacuolization of nuclei (arrow 5).

**Figure 2:**
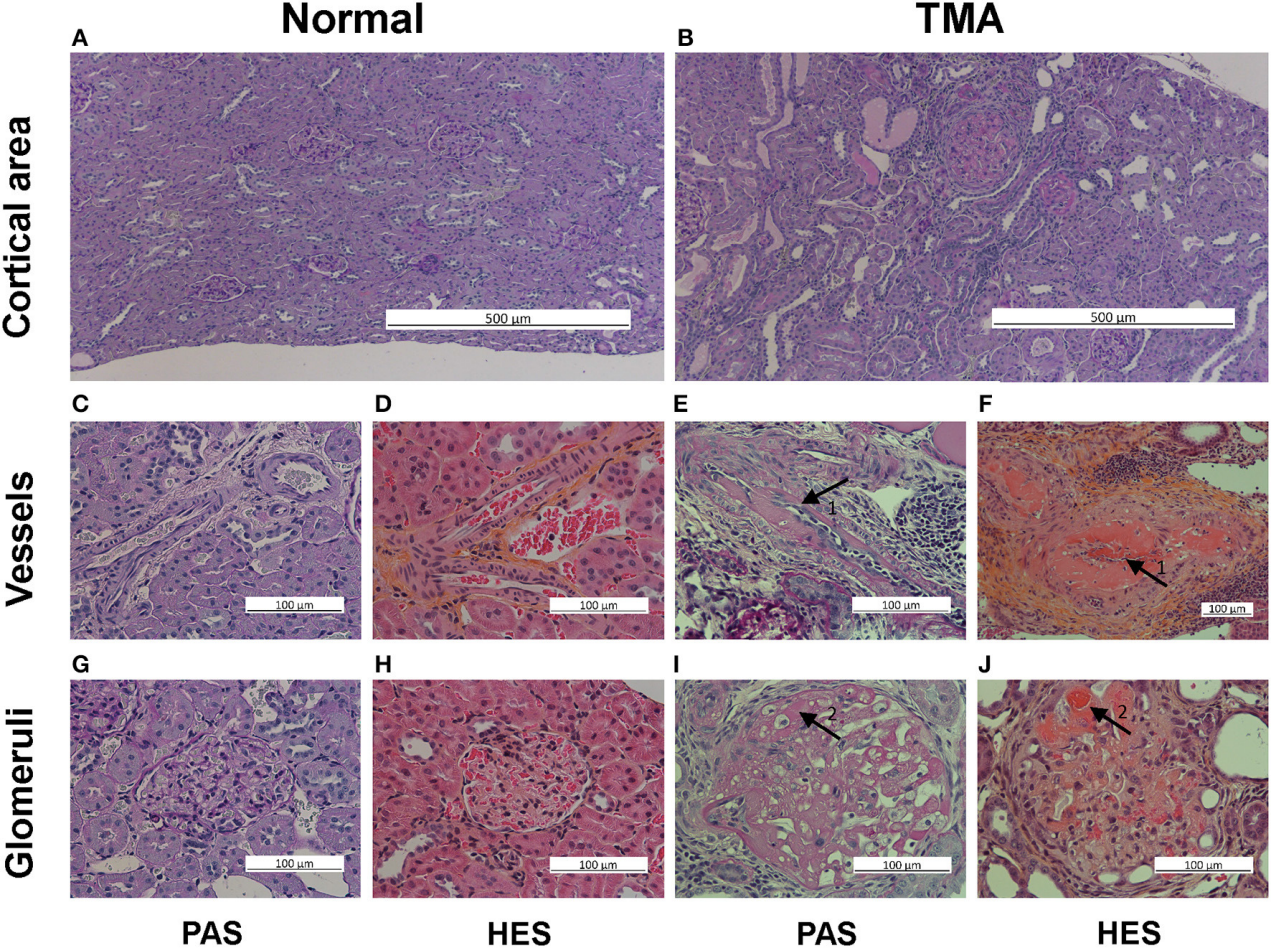
Thrombotic microangiopathy (TMA) histological assessment in PAS and HES staining, renal cortex. The top row illustrates normal tissue **(A)** and TMA **(B)** with protein filled atrophic tubular segments in the cortical area, low magnification, PAS staining. The middle row illustrates in high magnification normal vessels in PAS **(C)** and HES **(D)** staining, and partly thrombosed vessels (arrows 1) with fibrinoid necrosis of vessel wall in PAS **(E)** and HES **(F)** staining. The bottom row illustrates in high magnification normal glomerulus in PAS **(G)** and HES **(H)** staining and extensive micro-thrombi with fibrinoid necrosis (arrows 2) in PAS **(I)** and HES **(J)** staining.

Both brain and kidney specimens were harvested fresh at the end of the study. The brain and one kidney were fixed in 4% formaldehyde. The fixed brain and kidney specimen were sectioned in the sagittal plane, dehydrated, and paraffin wax embedded. Slides were cut at 5 μm thickness for hematoxylin-eosin-saffron and periodic acid-schiff staining. Histological examinations of brain and kidney were performed by two investigators blinded to diet and blood pressure. Hypertensive encephalopathy was characterized by posterior cerebral encephalopathy with histological loose ground mesh and vacuolization around the nuclei (Figure 1). Lesions were graded on a semi quantitative scale: zero (no changes), one (light changes), or two (heavy changes). Renal histology was performed by evaluating 45 consecutive glomeruli per slide. Glomeruli were classified by the presence or absence of thrombosed glomerular vessels and eventually the co-existence of vascular onion skin lesion or fibrinoid necrosis in vessel walls (Figure 2).

### Plasma miR Measurements

The miRCURY LNA miRNA plasma real-time quantitative PCR panel (Qiagen, Germany) was employed for c-miR quantification. We used a panel with 145 c-miRs chosen from a database with healthy and diseased individuals with different stages of various cancers, neurological, inflammatory, and other disorders, or based on the limited number of published studies available. The panel included five reference c-miRs stably expressed in plasma (miR-16, miR-103, miR-192, miR-93, and mir-451), two c-miRs to control for hemolysis (miR-451 and miR-23a-3p), an inter-plate calibrator in triplicate, and a negative control. For microRNA real-time qPCR, 7 μl RNA was reverse transcribed in 35 μl reactions using the miRCURY LNA™ Universal RT microRNA PCR (Exiqon, Denmark). cDNA was diluted 50x and assayed in 10 μl PCR reactions according to the protocol; each c-miR was assayed once by qPCR on the plasma focus panel using ExiLENT SYBR® Green master mix. The amplification was performed in a LightCycler® 480 real-time PCR system (Roche) in 384 well plates. The amplification curves were analyzed using the Roche LC software, both for determination of Cq (by the 2nd derivative method) and for melting curve analysis. The amplification efficiency was calculated using algorithms similar to the LinReg software. All assays were inspected for distinct melting curves and the Tm was checked to be within known specifications for the assay. Furthermore, assays must be detected with 3 Cqs less than the negative control and with Cq <37 to be included in the data analysis. Data that did not pass these criteria were omitted from any further analysis. All data were normalized to the average of all samples (average – assay Cq).

### Statistical Analyses

Animal survival in protocol (before termination) is given as average number of days, tested by the log rank test. Baseline clinical and laboratory characteristics are given as mean for normally distributed variables, median for other variables. Statistical significance between HS and LS groups was tested by *T*-test. Number of outcomes (HE, TMA, HFpEF, and ED) and premature death was tested using Pearson's chi-squared test. Facing information from a large number of c-miRs, we applied partial least squares discriminant analysis (PLS-DA) for dimensionality reduction and selection of important variables for discriminating animals with different hypertensive end-organ injuries from animal not experiencing such outcomes (36). c-miR values of zero (no detectable signal) were replaced by a small value or removed if >50% were missing (3–4% of all c-miRs for all outcomes). C-miRs output data were logarithmically transformed, mean-centered, and divided by the standard deviation of each variable before the statistical analysis. Variable importance in projection (VIP) was used to evaluate the importance of the variables. The VIP score is based on the sum of variable influence over all model dimensions and can be used to identify discriminating variables or predictors (37). Recommended VIP score cutoffs vary from 0.8 to 1.2 (38–42). We chose a cutoff >1.2 for our study. We used receiver operating characteristic (ROC) curves to evaluate the diagnostic accuracy of a composite outcome of ongoing hypertensive end-organ damage. Sensitivity and specificity are given for the top left corner of the ROC-curve (ROC 01). Benjamini-Hochberg adjustment was applied to control false discovery rate (FDR) caused by multiple comparisons (43), and a q-value <0.10 was used to select c-miRs with an area under the ROC curve (AUC) significantly different from 0.50. Diagnostic accuracy was quantified by the area under the ROC curve (AUC) and categorized as fair, good, or excellent (AUC 0.70–0.79, 0.80–0.89, and 0.90–1.00, respectively). Visualization and statistical testing of multiset intersections were done with the Super Exact Test R-package to yield more in-depth understanding of interactions and connections between c-miRs and the various hypertensive emergency outcomes (44). Statistical analyses of outcomes were performed using SPSS (V25.0, SPSS, Armonk, New York), and PLS-DA based analyses were performed with MetaboAnalyst 3.0 (42). For c-miR target prediction and pathway analysis we used the miRbase (version 21). miR: mRNA interactions are derived from miRNA target prediction algorithm (TargetScan) using DIANA Tools (43). The DIANA-miRPath v3.0 algorithm was used to enable the identification of pathways based on Kyoto encyclopedia of genes and genomes (KEGG) molecular pathways for *Rattus norvegicus*. For each outcome, all c-miRs with a VIP-score >1.2 served as input for pathway analysis. The identified pathways were then ranked by their *p*-value, and a common list of the top 20 pathways by their median rank was produced. In a sensitivity analysis, we selected c-miRs that exclusively identified one outcome and used these as input for pathway analysis.

## Results

### Hypertensive Outcomes

**Figure 3:**
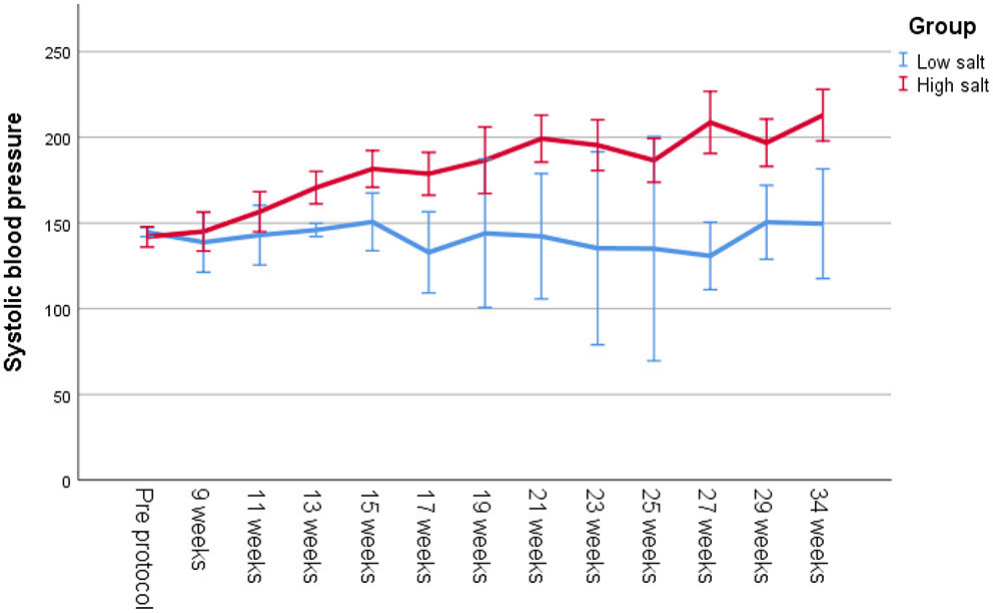
Systolic blood pressure measured with tail cuff on unanesthetized Dahl/SS on a low-salt vs. a high-salt diet throughout the study.

**Figure 4:**
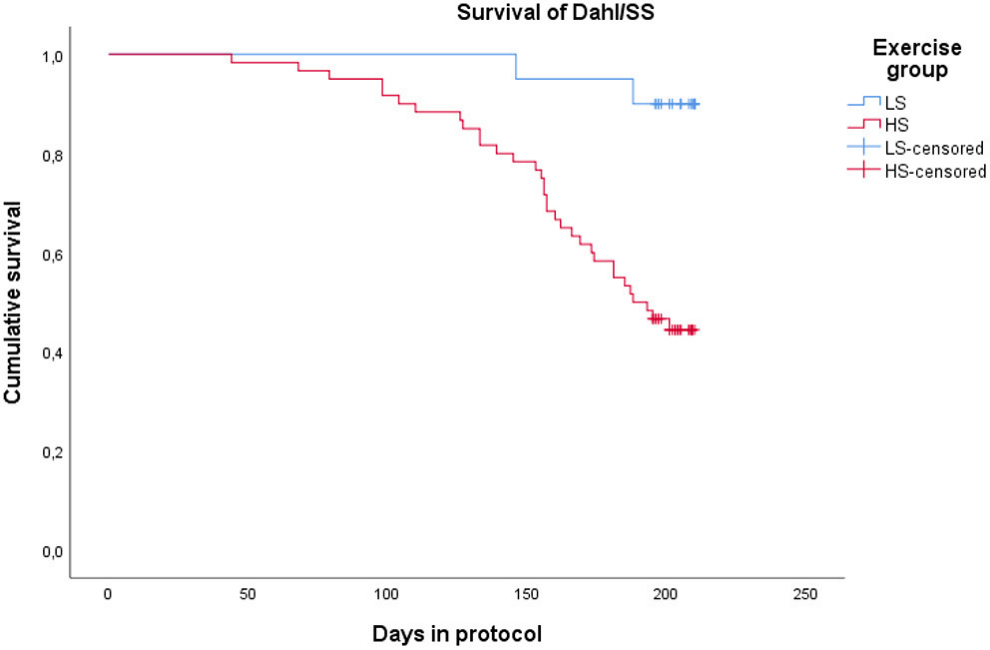
Kaplan-Meyer plot showing survival of Dahl/SS on a low-salt vs. a high-salt diet. Termination at the end of protocol is censored.

All Dahl/SS rats experienced increasing blood pressure over the study period (Figure 3). Rats on a high-salt diet had severe hypertension with a mean blood pressure of 205/147 mm Hg (95% CI 192–217/135–160 mm Hg) at the end of study, while low-salt animals had mild to moderate hypertension (152/108 mm Hg, 95% CI 137–167/94–122 mm Hg, *p* < 0.001 compared to HS group) (Table 1). Figure 4 shows that animals in the HS group on average survived 175 days in protocol vs. 206 days for the LS group (*p* = 0.001). Twenty-six of the 35 animals dying or being euthanized before the end of the protocol showed signs of stroke or severe HE. Among the remaining animals, the HS group had a strongly increased albumin excretion rate compared to the LS group, while there was only a slightly lower creatinine clearance, indicating early kidney damage. Circulating miRs were analyzed in 35/45 animals at the end of the experiment. Hypertensive end organ injuries were found mainly among the HS animals. HE was found in 10/19 HS vs. 0/12 LS animals at the end of study (*p* = 0.002). TMA of the kidneys was found in 12/20 HS vs. 0/15 LS animals (*p* < 0.001). HFpEF was found in 7/20 HS vs. 1/11 LS animals (*p* = 0.115). ED was found in 8/16 HS vs. 3/11 LS animals (*p* = 0.238). C-miRs were then analyzed for each outcome among all Dahl/SS, both LS and HS.

**Table 1:** Main clinical characteristics and outcomes for Dahl SS rats surviving to 29–30 weeks by diet (low-salt, 0.3% NaCl or high-salt, 8% NaCl).

**Clinical characteristics**	**LS (*n* = 7–15)**	**HS (*n* = 11–18)**	
Body weight, grams	294 (286–303)	285 (277–293)	0.102
Systolic blood pressure, mmHg	153 (135–171)	201 (188–213)	<0.001
Diastolic blood pressure, mmHg	108 (91–124)	144 (131–157)	<0.001
24 h albuminuria, mg/24 h	0.59 (0.29–1.10)	81 (23–149)	<0.001
Creatinine clearance, ml/min	1.9 (1.4–2.4)	1.7 (1.3–2.1)	0.514
**Hypertensive outcomes**	**LS (*****n*** **=** **11–15)**	**HS (*****n*** **=** **16–20)**	
Hypertensive encephalopathy	0	55	0.002
Thrombotic microangiopathy	0	62	<0.001
Heart failure with preserved EF	9	35	0.115
Endothelial dysfunction	27	50	0.238

*Clinical characteristics are mean with 95% CI (median for albuminuria), statistics by t-test. Hypertensive outcomes are presented as percentage of animals presenting each outcome among LS and HS animals, statistics by Chi-square*.

### c-miRs as Potential Biomarkers for Hypertensive Emergency Outcomes

**Figure 5:**
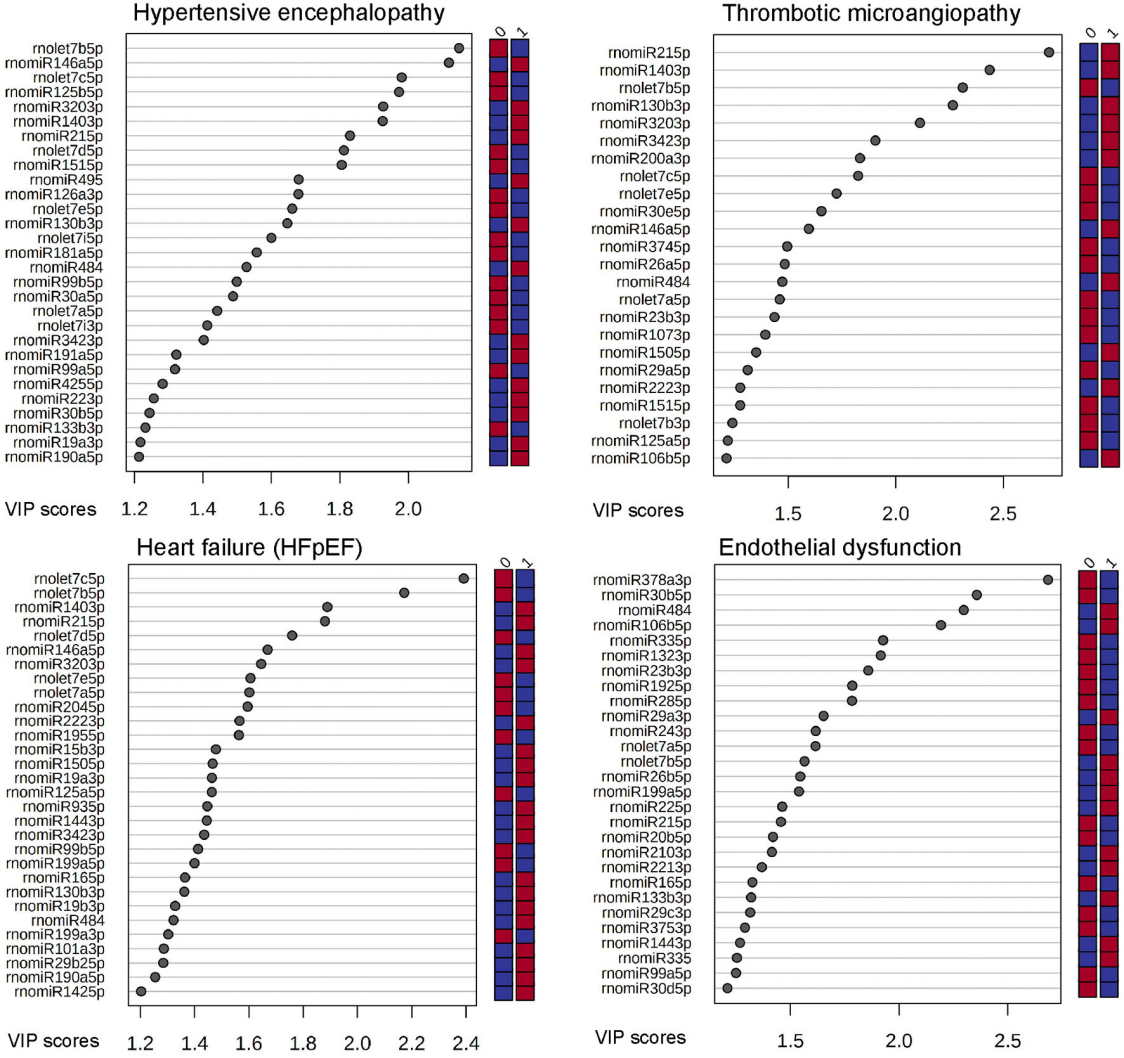
C-miRs ranged by their VIP-score (>1.2) by each hypertensive outcome. The right column indicates if cases (1) or controls (0) were up-regulated (red) or down-regulated (blue).

**Figure 6:**
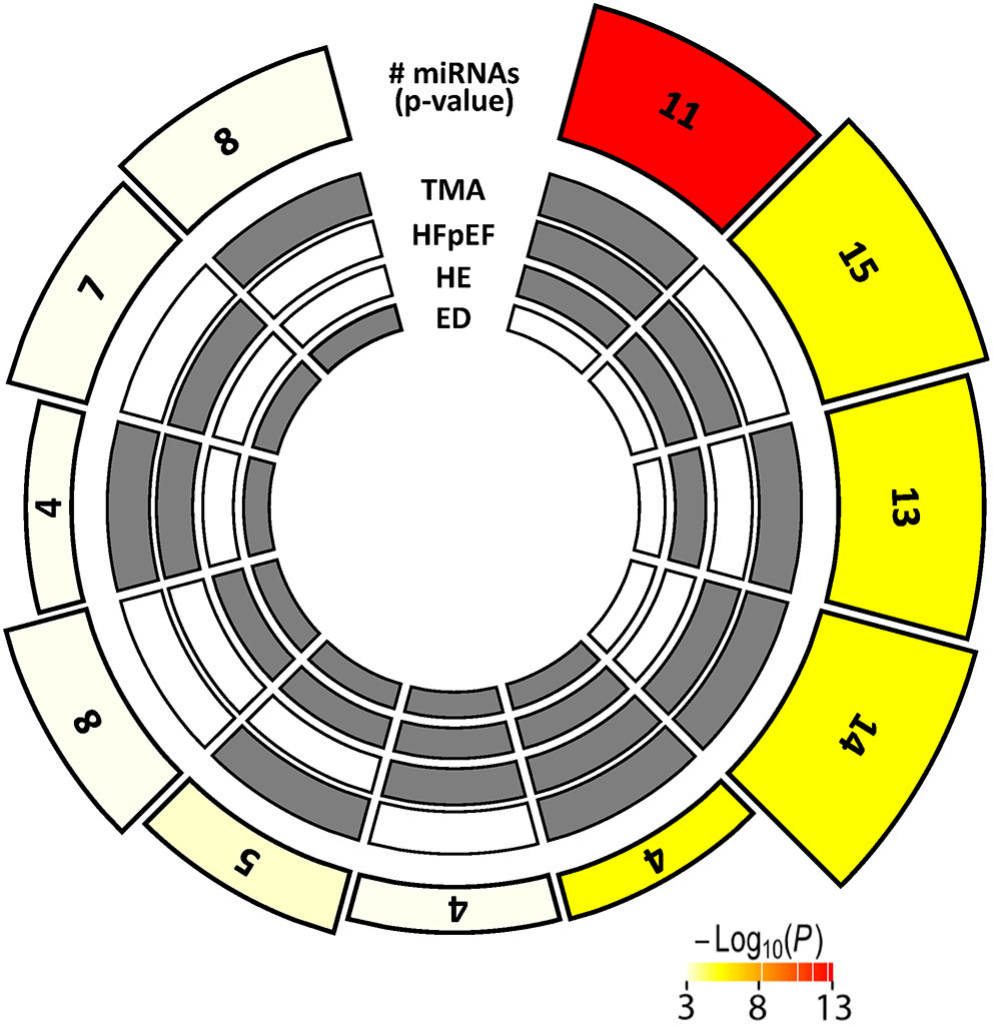
Multi-layer circular plot displays c-miR intersections for two or more of the hypertensive end-organ injuries studied. The four inner circles represent the presence (gray) or absence (white) of dysregulated c-miRs by the four different end-organ injuries, radially outwards: Endothelial dysfunction (ED), hypertensive encephalopathy (HE), heart failure with preserved ejection fraction (HFpEF), and thrombotic microangiopathy (TMA). The height of the bars on the outermost track is proportional to the number of intersecting c-miRs common among the given combination of hypertensive outcomes, and the fill color of the outermost bar color intensity represents the statistical significance of the intersection (*p* < 0.001 coded white).

Figure 5 displays c-miRs with high VIP-scores (>1.2) in the individual hypertensive outcomes, indicating important discrimination between the presence or absence of each outcome (29 c-miRs associated with HE, 24 c-miRs with TMA, 30 c-miRs with HFpEF, and 28 c-miRs with ED). Seventeen c-miRs were common for two outcomes in different combinations, seven c-miRs for three outcomes in different combinations, and four c-miRs were common for all four outcomes indicating common pathophysiology. Figure 6 depicts a circular plot illustrating all intersections of c-miRs associated with at least two outcomes and the corresponding statistics. ED differs from the other outcomes by having fewer and less significant interactions with the other outcomes and a larger proportion of c-miRs exclusively identifying this condition. All intersections of c-miRs between HE, HFpEF and TMA were highly significant (*p* < 0.001), indicating a common c-miR-associated pathophysiological mechanism. More details and the specific c-miRs included in the various intersections are given in Supplementary Tables 1, 2. A Venn diagram of all dysregulated c-miRs according to outcomes is given in Supplementary Figure 1. Supplementary Table 2 also shows 40 c-miRs that exclusively discriminate one single hypertensive outcome with a VIP-score >1.2, indicating separate pathophysiology (10 c-miRs only associated with HE, 6 c-miRs only associated with renal TMA, 9 c-miRs only associated with HFpEF, and 15 c-miRs only associated with ED). Table 2 shows ROC-curve AUC and *p*-values values for the diagnostic accuracy of various c-miRs for a composite endpoint of end-organ damage (combinations of HE, TMA, and/or HFpEF). Among these, there were four c-miRs with good accuracy (miR-21-5p, let-7b-5p, miR-140-3p, and miR-126a-3p) and six c-miRs with fair accuracy (miR-130b-3p, miR-200a-3p, let-7c-5p, miR-320-3p, miR-342-3p, and miR-27b-3p).

**Table 2:** List of c-miRs with significant AUC of the ROC-curve for a composite hypertensive outcome of HE, TMA, and/or HFpEF (displaying actual *p*-values selected by FDR *p*-value <0.10).

**c-miR**	**AUC**	**95% CI**	***p*-value**	**Sensitivity**	**Specificity**
rno-miR-21-5p	0.88	0.758–0.974	2.62E-5	0.83	0.88
rno-let-7b-5p	0.84	0.683–0.971	5.41E-4	0.83	0.82
rno-miR-140-3p	0.84	0.698–0.955	6.46E-4	0.67	0.94
rno-miR-130b-3p	0.79	0.618–0.927	0.003	0.78	0.77
rno-miR-200a-3p	0.78	0.578–0.922	0.004	0.67	0.82
rno-let-7c-5p	0.76	0.595–0.900	0.006	0.72	0.71
rno-miR-320-3p	0.75	0.562–0.910	0.006	0.61	0.82
rno-miR-126a-3p	0.81	0.657–0.944	0.007	0.78	0.77
rno-miR-342-3p	0.74	0.567–0.895	0.01	0.67	0.71
rno-miR-27b-3p	0.75	0.580–0.904	0.01	0.72	0.77

*Sensitivity and specificity are given for the top left corner of the ROC-curve*.

### KEGG Molecular Pathways Associated With c-miRs

Table 3 lists the top 15 pathways for the four outcomes by their median rank. Several important signaling pathways with clear relevance to hypertension and cardiovascular disease were discovered. Mucin type O-glycan biosynthesis pathway had the overall highest rank being number one in HE (*p* = 3.9E-09), number one in TMA (*p* = 1.7E-05), and second in HFpEF (*p* = 3.3E-07). The MAPK signaling pathway was ranked very high in TMA, HFpEF, and ED (*p* < 0.0001 in all). Other signaling pathways such as FoxO, Wnt, Hippo, and TGF-beta were also significant (*p* < 0.01 for all) and highly ranked in all types of hypertensive emergency complications.

**Table 3:** KEGG pathways identified by c-miRs associated with two or more organ damages sorted by their overall (median) rank.

**Overall rank**	**KEGG pathway**	**HE**		**TMA**		**HFpEF**		**ED**	
		***p*-value**	**Rank**	***p*-value**	**Rank**	***p*-value**	**Rank**	***p*-value**	**Rank**
1	Mucin type O-Glycan biosynthesis	3.9E-09	1	1.7E-05	1	3.3E-07	2	NR	
2	MicroRNAs in cancer	5.5E-04	4	1.7E-05	2	1.1E-09	1	1.2E-13	1
3	MAPK signaling pathway	1.8E-02	17	1.4E-04	3	4.8E-07	3	5.6E-06	3
4	Proteoglycans in cancer	1.0E-05	2	2.7E-03	20	8.9E-07	4	2.3E-04	7
5	Glioma	2.3E-03	7	1.3E-03	6	5.7E-03	18	4.4E-08	2
6	Pathways in cancer	4.7E-03	10	1.0E-03	5	2.4E-04	10	5.6E-05	5
7	Renal cell carcinoma	2.2E-02	22	1.8E-03	8	1.2E-04	8	1.6E-05	4
8	FoxO signaling pathway	2.0E-03	5	1.0E-03	4	7.8E-04	13	2.7E-03	15
9	Glycosaminoglycan biosynthesis—KS	NR		1.8E-03	7	NR		2.7E-03	11
10	Neurotrophin signaling pathway	7.6E-03	11	1.8E-03	11	7.6E-05	6	5.2E-04	9
11	Wnt signaling pathway	5.3E-04	3	1.8E-03	12	1.3E-04	9	5.6E-03	21
12	Stem cells pluripotency signaling pathways	2.3E-03	8	2.0E-03	16	4.2E-05	5	8.1E-03	25
13	Synaptic vesicle cycle	NR		1.8E-03	9	5.4E-04	12	3.1E-02	32
14	Hippo signaling pathway	2.0E-03	6	2.2E-03	19	3.0E-04	11	4.3E-03	18
15	TGF-beta signaling pathway	2.5E-03	9	2.0E-03	15	2.6E-03	14	4.3E-03	17

*Based on pathway lists for each single hypertensive outcome from c-miRs with VIP-scores >1.2. p-values are FDR adjusted. NR, not ranked; KS, keratin sulfate; ER, endoplasmatic reticulum*.

Table 4 lists the most important pathways identified using c-miRs exclusively associated with only one of the outcomes. Fatty acid degradation was identified by both HE and HFpEF (HE; *p* = 9.4E-07 and HFpEF; *p* = 5.4E-10). This was also the case for Fatty acid metabolism (HE; *p* = 0.004 and HFpEF; *p* = 3.3E-06).

**Table 4:** Additional KEGG pathways identified by list of exclusive c-miRs for each hypertensive outcome.

**KEGG pathways**	***p*-value**	**Outcomes**
Fatty acid degradation	9.4E-07 5.4E-10	HE HFpEF
Fatty acid metabolism	0.004 3.3E-06	HE HFpEF
ECM-receptor interaction	1.9E-22	ED
Glycosphingolipid biosynthesis—lacto and neolacto series	9.6E-05	HE
Other glycan degradation	0.0002	HE
Vitamin digestion and absorption	0.0006	HE
Purine metabolism	0.005	TMA

C-miRs VIP-scores >1.2. Pathways ranked by their p-value.

*HE, hypertensive encephalopathy; TMA, thrombotic microangiopathy; HFpEF, heart failure with preserved ejection fraction; ED, endothelial dysfunction*.

## Discussion

We found 68 c-miRs significantly associated with hypertensive emergency complications in an animal model of severe hypertension. Twenty-eight c-miRs identified more than one outcome, and 11 comprised a common signature for early organ damage of the brain, kidney, and heart. ROC analysis revealed four c-miRs with good and six c-miRs with fair identification of composite early end-organ injury. Panels of c-miRs identified by principal component analysis showed significant association with several signaling pathways of importance for cardiovascular disease. Although several c-miRs were associated with endothelial dysfunction, our findings did not support the hypothesis that ED through circulating microRNA act as a mediator of hypertensive emergency end-organ injury in the brain, kidney, and heart.

The pathogenesis in hypertensive emergencies is not fully understood, but rapidly increased blood pressure caused by the failure of arteriolar autoregulation is postulated to cause ED, renin-angiotensin-aldosterone system activation, and a pro-thrombotic state leading to organ injury (1, 45). We found deviations in the levels of c-miRs in animals suffering end-organ injuries, raising the possibility that miRs are involved in the hypertensive emergency pathogenesis. c-miRs may also present prognostic value as biomarkers in hypertensive emergency, as was recently highlighted in primary hypertension and endothelial dysfunction (46). The physical forces of the high blood pressure in hypertensive emergency contribute to endothelial cell dysfunction itself, but the initial injury of the vessel wall may also be caused by oxidative stress and inflammation. Endothelial dysfunction then starts a viscous circle with vascular smooth muscle cell (VSMC) dedifferentiation, proliferation, contraction, and vessel stiffening. Previous studies have demonstrated that miRs play a key part in these pathological processes by affecting VSMCs and endothelial cells (47–49). Several flow-sensitive miRs with both pro- and anti-atherogenic effects have already been identified (miR-126, miR-10a, miR-19a, miR-23b, miR-21, miR-663, miR-92a, miR-143, miR-101, miR-712, miR-205, miR-155, and miR-146a) (48). Five of these miRs were found significantly associated with ED in our study, corroborating its important role in severe hypertension. More specifically, miR-21, miR-146a, as well as miR-221, miR-222, miR-132, and miR-133, all found differently expressed in our study, have been demonstrated to control VSMC proliferation and dedifferentiation (47).

HE has long been considered as one of the major clinical manifestations of hypertensive emergency. The underlying mechanism is postulated to be caused by hypertension exceeding the upper limit of cerebral blood flow autoregulation, breakdown of the blood-brain barrier, and extravasation of plasma and macromolecules (50). Currently, there are no published studies on the involvement of miRs in the pathogenesis of HE. However, miR findings in patients with stroke might be of relevance since hypoperfusion, endothelial dysfunction, and minute as well as larger foci of hemorrhagic and ischemic lesions are also found in patients with posterior reversible encephalopathy syndrome (PRES) induced by severe hypertension (50, 51). Stroke patients display platelet activation, blood coagulation and inflammation (52, 53), and a recent bioinformatics analysis based on 25 studies showed that miR-19a-5p could be the central regulator of these three processes (54). Interestingly, miR-19a-5p was also selected as an important biomarker of endothelial dysfunction and hypertensive encephalopathy in our study. MiR-19a and MiR-19b have been shown to be associated with different cardiac diseases and generally promote proliferation and inhibit apoptosis. The MiR-19b family of microRNAs inhibit angiogenesis and promote apoptosis of endothelial cells, but seem to have opposing effects in different settings, exemplified by promotion of angiogenesis in tumor cells. Furthermore, the miR-19 family plays a role in differentiation and migration of neurons, as well as participating in the regulation of both neurodegenerative diseases and neuronal neoplasms. Of interest, miR-19b is upregulated in neural progenitor cells in human stroke (55). There are evidences indicating that miR-19a stimulates arteriogenesis and reduce apoptosis in endothelial cells, and it also seems to have a role in the differentiation, cell survival, and migration of neurons (55). Furthermore, miR-15, miR-16, and miR-129 were found to regulate blood coagulation and platelet activation in stroke (55), and miR-16 and miR-129 were also found dysregulated in our study indicating that hypertensive encephalopathy and ischemic stroke could share some common pathophysiological mechanisms.

Recent studies indicate that complement activation, inflammation, and oxidative stress cause endothelial injury in addition to the hypertension induced shear forces (56). It therefore seems to be considerable overlap between microangiopathies caused by severe hypertension, atypical hemolytic uremic syndrome (aHUS), and thrombotic thrombocytopenic purpura (57), and miR based studies from the two latter diseases could therefore be of importance for understanding TMA. For instance, patients with Shiga-toxin induced HUS present with increased levels of miR-24 and miR-126 possibly reflecting endothelial injury (58). In addition, HUS patients display changes in four other miRs (miR-200, miR-150, miR-26a, miR-30e), all associated with renal TMA in our study.

Hypertension is the most frequent comorbidity found in patients with HFpEF, whereas patients with HFrEF more often have a history of ischemic heart disease (59, 60). Many studies describe important changes in circulating miRs for patients with HFrEF (e.g., miR-7, miR-21, miR-22, miR-26, miR-27, miR-29, miR-30, miR-133a, miR-214, miR-301) (61, 62), while less is known about c-miRs and HFpEF (63). The underlying mechanisms of HFpEF are incompletely understood. Traditionally, increased afterload with compensatory myocyte hypertrophy leading to maladaptive left ventricle hypertrophy and diastolic dysfunction has been the main explanatory model. The new paradigm of HFpEF pathophysiology stress the importance of comorbidities causing an early systemic inflammatory state. Endothelial dysfunction with increased production of reactive oxygen species limits nitric oxide availability in adjacent cardiomyocytes. This in turn decreases activity in protein kinase G and removes the brake on cardiomyocyte hypertrophy, leading to concentric left ventricular remodeling (60). Increased myofibroblast collagen deposition also contributes to diastolic dysfunction (60, 64–66). Our c-miR findings might be associated with fibrosis, inflammation, and energy imbalance (67). HFpEF animals in our study had reduced levels of let-7c and miR-125a, which has been linked to promoting an anti-inflammatory phenotype of macrophages (68). Additionally, miR-146a and miR-21 were upregulated, and have been associated with energy imbalance (69) and cardiac fibrosis (18, 70), respectively. Other studies have found miR-378 to play an important role in fatty acid metabolism, regulating the metabolic fuel shift in cardiomyocytes (71), but circulating levels were not affected by a diagnosis of HFpEF in our rats. On the other hand, miR-378 turned out to be the most important microRNA in discriminating animals with ED in our study. The metabolic shift seems to be of importance also among the c-miRs that exclusively identified each outcome, where fatty acid metabolism and fatty acid degradation were the most important HFpEF pathways.

MiRs are known to modulate multiple targets, so pathway analysis can be useful to understand the overall biological effects. To this end, mucin-type O-glycan pathway was found strongly enriched in HE, TMA, and HFpEF. In fact, other studies have also found aberrant glycosylation in the left ventricle and plasma of Dahl/SS rats on a HS diet associated with cardiac hypertrophy and heart failure (72). Glycosylation, the post-translational addition of carbohydrate chains to proteins, is important to protect and modify the function of structural proteins and enzymes (73, 74). O-glycans are abundantly present on all cell surfaces, and more specifically, the endothelial glycocalyx physically protects the vessel wall and modulate interactions with the environment and other cells (75). Glycocalyx injury can be caused by physical forces (locally reduced shear stress due to lack of laminar flow, hypervolemia, or hypertension) and chemical degradation (inflammatory mediators and reactive oxygen species induced by sepsis, ischemia reperfusion, smoking, etc.) (76–78). This leads to reduced NO-mediated vasodilatation, increased endothelial permeability, and comprehensive hemostatic disturbances, which can both initiate, progress, and complicate cardiovascular disease (79, 80). Studies on von Willebrand factor have revealed extensive glycosylation of the protein structure, and this regulates the susceptibility to proteolysis (81). Disturbances in the glycocalyx and von Willebrand factor clearly can facilitate hypertensive emergency complications like thrombotic microangiopathy. In addition, hypertension is associated with reduced glycocalyx in both rodents and humans (82, 83), but the underlying mechanism is not fully elucidated. High blood pressure is associated with increased levels of sodium, aldosterone, and atrial natriuretic factor in the circulation, and each of these are known to directly impair the glycocalyx (84). There are no other studies published on c-miRs associated with hypertensive emergencies.

We also found that the MAPK signaling pathway was highly associated with hypertensive emergencies outcomes, and although there are no studies in patients with hypertensive emergencies, several reports bring support to our findings (10, 85–87). Angiotensin II activates its receptor on the endothelial cell surface triggering formation of reactive oxygen species and activation of several intracellular pathways like MAPK (ERK1/2) and others (Akt, NF-kB, MMPs) (87). A large number of transcription factors are then activated or repressed leading to fibroblast-to-myofibroblast activation, myocyte growth, vascular smooth muscle cell phenotype switching, inflammation, and fibrosis (88, 89). In addition to neurohumoral activation, high blood pressure causes direct mechanical stress which can activate the MAPK pathway *via* various cell-surface proteins (85). p38-MAPK-inhibitors can attenuate hypertensive target organ injury in rat models (10, 86). Therefore, MAPK pathway signaling is strongly involved in the development of cardiac hypertrophy and vascular stiffening, and our data seem to extend this to end-organ injury in hypertensive emergencies.

### Strengths and Weaknesses

In our experiment, we used an established animal model of salt-sensitive hypertension to provoke typical end-organ injuries of hypertensive emergency. Our model produced a fair number of hypertensive end-organ injuries, and thereby making it a good model for exploratory search of potential biomarkers and pathways involved. A limitation could be the use of female rats only, as both genders ideally should be studied. There are currently no data advocating major gender differences in hypertensive emergency end-organ injuries (7, 13). HFpEF as a chronic complication of hypertension is more common in elderly women (90). We used a well-validated miR panel for biofluids with thorough quality control assessment. However, we have screened only 145 out of thousands of miRs in the circulation, limiting our findings to deviations among the miRs investigated. An individual c-miR may be dysregulated by quite a few different conditions, thereby being a non-specific biomarker of disease, whereas combinations of c-miRs' expressions may act as a more precise diagnostic tools in a setting where different conditions are considered.

The cross-sectional design of the study has more limitations compared to a longitudinal cohort. MiRs occur at very low levels in the circulation and their association to disease is less well-established compared to tissue-miRs. There is also a lack of house-keeping miRs needed for normalization of results which together with the lack of standardization of kits from different suppliers impairs comparison of results from different studies. Pre-analytical factors like hemolysis and cell injury can be problematic in miR studies, but this was compensated for in our panel by including c-miRs for reference genes as well as c-miRs for hemolysis. Our findings need confirmation in a confirmation cohort.

## Conclusions

Our animal model of severe hypertension demonstrated brain, kidney, and heart end-organ injury compatible with human hypertensive emergency. We found a number of circulating microRNAs associated with combined end-organ injury, as well as c-miRs exclusively associated with individual outcomes. HE, TMA, and HFpEF were more often associated with the same c-miRs than ED, indicating common pathophysiology of the former three. Disturbances in important pathways related to cardiovascular disease were associated with the end-organ injuries of hypertensive emergency in our rat model. Circulating miRs could have a potential for early diagnosis of end-organ injury in hypertensive emergency, requiring urgent treatment. Pathway prediction tools also elucidate possible mechanisms in hypertensive emergency, that may be subject for further investigations.

## Data Availability Statement

The raw data supporting the conclusions of this article will be made available by the authors, without undue reservation.

## Ethics Statement

The animal study was reviewed and approved by the Norwegian Regulation on Animal Experimentation.

## Author Contributions

VA, UW, and SH conceived and designed research. KL, GS, TO, and NR performed experiments. KL, GS, TO, NR, and SH analyzed data. KL, GS, HD, and SH interpreted results of experiments. KL, GS, and SH drafted manuscript. All authors revised and approved the manuscript.

## Conflict of Interest

The authors declare that the research was conducted in the absence of any commercial or financial relationships that could be construed as a potential conflict of interest.
