# Supplementary figures and images for: Circulating microRNAs May Serve as Biomarkers for Hypertensive Emergency End-Organ Injuries and Address Underlying Pathways in an Animal Model
Source: Front Cardiovasc Med. 2021 Feb 12;7:626699. doi: 10.3389/fcvm.2020.626699 (PMC7906971; doi:10.3389/fcvm.2020.626699)

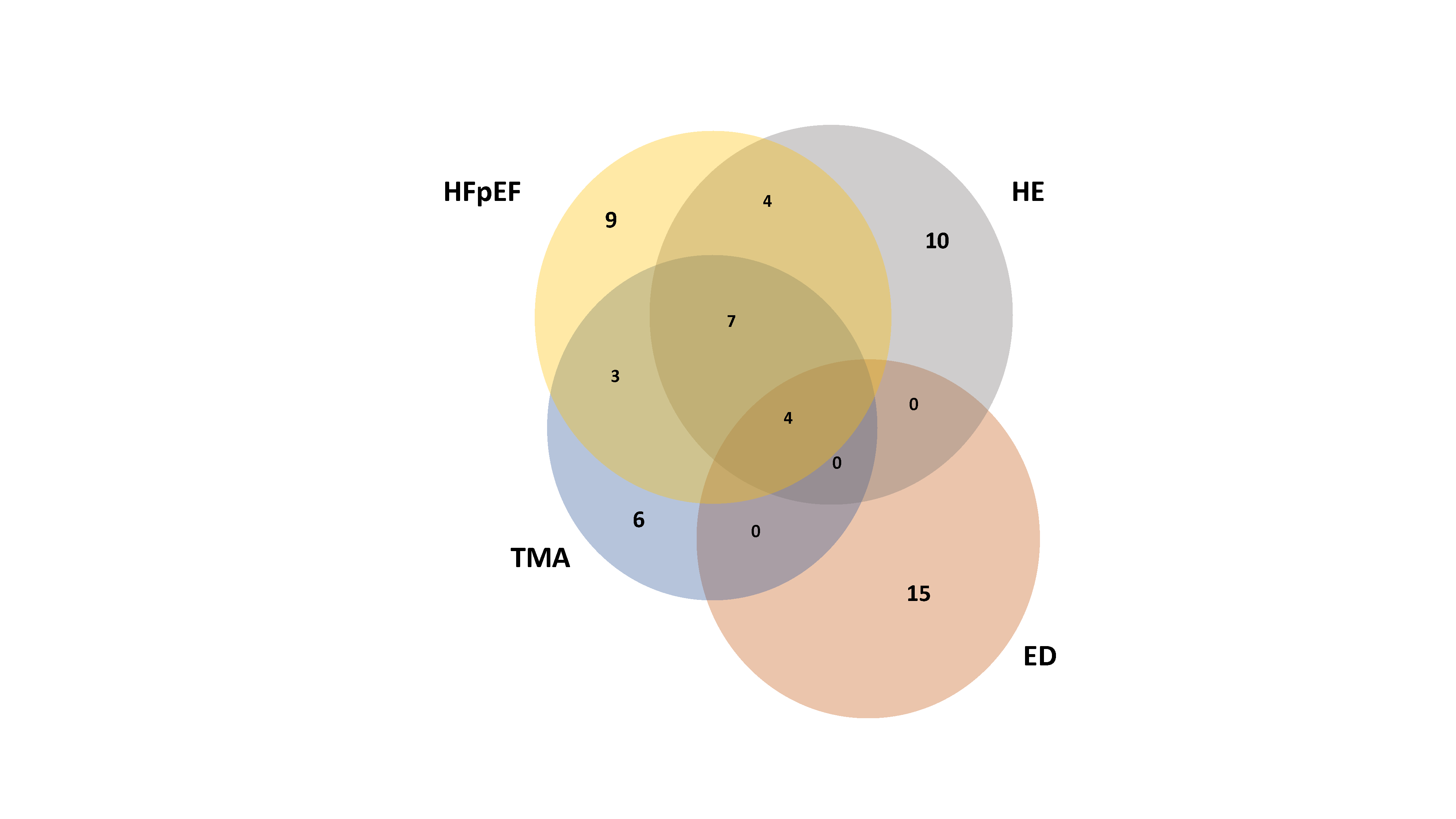

Supplement: Supplementary file 1 [file Image_1.TIF]
