# Supplementary material for: Circulating microRNAs May Serve as Biomarkers for Hypertensive Emergency End-Organ Injuries and Address Underlying Pathways in an Animal Model
Source: Front Cardiovasc Med. 2021 Feb 12;7:626699. doi: 10.3389/fcvm.2020.626699 (PMC7906971; doi:10.3389/fcvm.2020.626699)
Supplement: Supplementary file 2 [file Table_1.PDF]

Supplementary table S1. Most important variables for predicting hypertensive outcomes based on VIP scores from discriminant analysis of c-miRs in alphabetic order**.**

**c-miR**	**Hypertensive outcome**			
	**HE**	**TMA**	**HFpEF**	**ED**
rno-let-7a-5p	1.44	1.46	1.60	1.62
rno-let-7b-3p		1.24		
rno-let-7b-5p	2.15	2.31	2.17	1.57
rno-let-7c-5p	1.98	1.82	2.39	
rno-let-7d-5p	1.81		1.76	
rno-let-7e-5p	1.66	1.72	1.61	
rno-let-7i-3p	1.41			
rno-let-7-i5p	1.60			
rno-miR-15b-3p			1.48	
rno-miR-16-5p			1.36	1.33
rno-miR-19a-3p	1.23		1.46	
rno-miR-19b-3p			1.33	
rno-miR-20b-5p				1.42
rno-miR-21-5p	1.83	2.71	1.88	1.46
rno-miR-22-3p	1.26			
rno-miR-22-5p				1.46
rno-miR-23b-3p		1.44		1.86

rno-miR-24-3p				1.62
rno-miR-26a-5p		1.48		
rno-miR-26b-5p				1.55
rno-miR-28-5p				1.78
rno-miR-29a-5p		1.31		1.65
rno-miR-29b-5p			1.28	
rno-miR-29c-3p				1.32
rno-miR-30a-5p	1.49			
rno-miR-30b-5p	1.24			2.36
rno-miR-30d-5p				1.21
rno-miR-30e-5p		1.65		
rno-miR-33-5p				1.93
rno-miR-93-5p			1.45	
rno-miR-99a-5p	1.32			1.25
rno-miR-99b-5p	1.50		1.41	
rno-miR-101a-3p			1.29	
rno-miR-106b-5p		1.21		2.19
rno-miR-107-3p		1.39		
rno-miR-125a-5p		1.23	1.46	

rno-miR-125b-5p	1.97			
rno-miR-126a-3p	1.68			
rno-miR-130b-3p	1.65	2.27	1.36	
rno-miR-132-3p				1.92
rno-miR-133b-3p	1.23			1.32
rno-miR-140-3p	1.93	2.4363	1.89	
rno-miR-142-5p			1.20	
rno-miR-144-3p			1.45	1.27
rno-miR-146a-5p	2.12	1.60	1.67	
rno-miR-150-5p		1.35	1.47	
rno-miR-151-5p	1.81	1.28		
rno-miR-181a-5p	1.56			
rno-miR-190a-5p	1.21		1.25	
rno-miR-191a-5p	1.32			
rno-miR-192-5p				1.78
rno-miR-195-5p			1.56	
rno-miR-199a-3p			1.30	

rno-miR-199a-5p			1.40	1.54
rno-miR-200a-3p		1.83		
rno-miR-204-5p			1.60	
rno-miR-210-3p				1.42
rno-miR-221-3p				1.37
rno-miR-222-3p		1.28	1.57	
rno-miR-320-3p	1.93	2.11	1.65	
rno-miR-335				1.25
rno-miR-342-3p	1.40	1.91	1.44	
rno-miR-374-5p		1.49		
rno-miR-375-3p				1.29
rno-miR-378a-3p				2.69
rno-miR-425-5p	1.28			
rno-miR-484	1.53	1.47	1.32	2.30
rno-miR-495	1.68			

Note: Table displays miRs with PLS-DA VIP-scores >1.2. HE, hypertensive encephalopathy; TMA, thrombotic microangiopathy; HFpEF, heart failure with preserved ejection fraction; ED, endothelial dysfunction.
