# Supplementary material for: Circulating microRNAs May Serve as Biomarkers for Hypertensive Emergency End-Organ Injuries and Address Underlying Pathways in an Animal Model
Source: Front Cardiovasc Med. 2021 Feb 12;7:626699. doi: 10.3389/fcvm.2020.626699 (PMC7906971; doi:10.3389/fcvm.2020.626699)
Supplement: Supplementary file 3 [file Table_2.PDF]

Supplementary Table S2. Systematic combinations of hypertensive outcomes and their associated c-miRs of substantial importance for discrimination between disease and non-disease.

**Hypertensive outcomes**				**N**	**c-miRs**
**ED**	**HE**	**TMA**	**HFpEF**		
*	*	*	*	4	rno-let-7a-5p, rno-let-7b-5p, rno-miR-21-5p, and rno-miR-484
*	*	*		0	---
*	*		*	0	---
*		*	*	0	---
	*	*	*	7	rno-let-7c-5p, rno-let-7e-5p, rno-miR-130b-3p, rno-miR-140-3p, rno-miR-146a-5p, rno-miR-320-3p, and rno-miR-342-3p
*	*			3	rno-miR-30b-5p, rno-miR-99a-5p, and rno-miR-133b-3p
*		*		3	rno-miR-23b-3p, rno-miR-29a-5p, and rno-miR-106b-5p
*			*	3	rno-miR-16-5p, rno-miR-144-3p, and rno-miR-199a-5p
	*	*		1	rno-miR-151-5p
	*		*	4	rno-let-7d-5p, rno-miR-19a-3p, rno-miR-99b-5p, and rno-miR-190a-5p
		*	*	3	rno-miR-125a-5p, rno-miR-150-5p, and rno-miR-222-3p,
*				15	rno-miR-20b-5p, rno-miR-22-5p, rno-miR-24-3p, rno-miR-26b-5p, rno-miR-28-5p, rno-miR-29c-3p, rno-miR-30d-5p, rno-miR-33-5p, rno-miR-132-3p, rno-miR-192-5p, rno-miR-210-3p, rno-miR-221-3p, rno-miR-335, rno-miR-375-3p, and rno-miR-378a-3p
	*			10	rno-let-7i-3p, rno-let-7i-5p, rno-miR-22-3p, rno-miR-30a-5p, rno-miR-125b-5p, rno-miR-126a-3p, rno-miR-181a-5p, rno-miR-191a-5p rno-miR-425-5p, and rno-miR-495
		*		6	rno-let-7b-3p, rno-miR-26a-5p, rno-miR-30e-5p, rno-miR-107-3p, rno-miR-200a-3p, and rno-miR-374-5p

			*	9	rno-miR-15b-3p, rno-miR-19b-3p, rno-miR-29b-5p, rno-miR-93-5p, rno-miR-101a-3p, rno-miR-142-5p, rno-miR-195-5p, rno-miR-199a-3p, and rno-miR-204-5p

Note: Hypertensive outcomes: HE, hypertensive encephalopathy; TMA, thrombotic microangiopathy; HFpEF, heart failure with preserved ejection fraction; ED, endothelial dysfunction; N, the number of c-miRs for the given combination of hypertensive outcomes.
